# The clinical implications of G1-G6 transcriptomic signature and 5-gene score in Korean patients with hepatocellular carcinoma

**DOI:** 10.1186/s12885-018-4192-1

**Published:** 2018-05-18

**Authors:** Sung-Min Ahn, Farhan Haq, Inkeun Park, Jean-Charles Nault, Jessica Zucman-Rossi, Eunsil Yu

**Affiliations:** 1 Department of Genome Medicine and Science, College of Medicine, Gachon University, Seongnam, South Korea; 2 Department of Hematology-Oncology, Gachon University Gil Hospital, Incheon, South Korea; 3 Department of Biosciences, Cancer Genetics and Epigenetics Lab, COMSATS Institute of Information Technology, Islamabad, Pakistan; 4 Inserm, UMR-1162, Génomique Fonctionnelle des Tumeurs Solides, Équipe Labellisée Ligue Contre le Cancer, 27 rue Juliette Dodu, F-75010 Paris, France; 5 Labex Immuno-Oncology, Sorbonne Paris Cité, Faculté de Médecine, Université Paris Descartes, Paris, France; 6 Sorbonne Paris Cité, UFR SMBH, Université Paris 13, F-93000 Bobigny, France; 7 Université Paris Diderot, F-75013 Paris, France; 8 Department of Pathology, University of Ulsan College of Medicine, Asan Medical Center, 88, OLYMPIC-RO 43-GIL, SONGPA-GU, SEOUL 138-736 South Korea

**Keywords:** 5-gene score, G1-G6 subgroups, Survival, Prognosis

## Abstract

**Background:**

Efforts have been made to classify Hepatocellular Carcinoma (HCC) at surgically curable stages because molecular classification, which is prognostically informative, can accurately identify patients in need of additional early therapeutic interventions. Recently, HCC classification based French studies on the expression of 16 genes and 5 genes were proposed. In 16-gene classification, transcriptomic signatures (G1-G6) were used to classify HCC patients into clinical, genomic and pathway-specific subgroups. In 5-gene score classification, the good or poor prognosis of HCC patients was predicted. The patient’s cohort in these studies was mainly from Caucasian and African populations. Here, we aimed to validate G1-G6 and 5-gene score signatures in 205 Korean HCC patients since genomic profiles of Korean patients are distinct from other regions.

**Methods:**

Integrated analyses using whole-exome sequencing, copy number variation and clinical data was performed against these two signatures to find statistical correlations. Kaplan-Meier, univariate and multivariate COX regression analysis were performed for Disease-Specific Survival (DSS) and Recurrence-Free Survival (RFS).

**Results:**

The G2 and G3 subgroups of transcriptomic signature were significantly associated with *TP53* mutations while G5 and G6 subgroups were significantly associated with *CTNNB1* mutations which is in concordance with original French studies. Similarly, the poor prognosis group of 5-gene score showed shorter DSS (*p* = 0.045) and early RFS (*p* = 0.023) as well as a significant association with microvascular invasion, tumor size (> 5 cm), elevated AFP levels, and *RB1* mutations. However, the 5-gene score was not an independent prognostic factor for survival.

**Conclusion:**

The G1-G6 and 5-gene signatures showed significant concordance between genetic profiles of Korean HCC patients and patients in original French studies. Thus, G1-G6 and 5-gene score signatures can be targeted as potential therapeutic biomarkers against HCC patients worldwide.

## Background

Hepatocellular carcinoma is the most common type of liver cancer worldwide [1]. Liver resection is one of the most viable treatment option for HCC patients, but associated with high risk of recurrence [2, 3]. In advanced HCC, no clinical trial studies have convincingly improved survival, except for the sorafenib trial [4]. The failure of these trials is partly due to the lack of effective molecular markers or the minimal validation of known molecular markers in diverse multi-ethnic populations. Efforts have been made to classify HCC at surgically curable stages because molecular classification, which is prognostically informative, can accurately identify patients in need of additional early therapeutic interventions [5–9].

In HCC, several molecular classification-based microarray studies have been reported. In the first two studies, HBV-positive HCC patients from Belgium and China were classified into good or poor prognosis groups [10, 11]. The expression of hepatoblast-related genes was significantly associated with poor prognosis of HCC patents [11]. In another HCC study using a Caucasian population of HCC patients, a gene signature of 186 genes was identified in non-tumor liver, out of which 113 showed good prognosis and 73 showed poor prognosis [12].

Recently, HCC classification methods based on the expression of 16 genes and 5 genes were proposed [13, 14]. In the 16-gene based classification, transcriptomic signatures (G1-G6) were used to classify HCC patients into clinical, genomic and pathway-specific G1-G6 subgroups [13]. In the 5-gene score-based classification, the good or poor prognosis of HCC patients was predicted [14]. The study showed the efficacy of 5-gene score as a potential biomarker in American, Caucasian and Chinese HCC patients [14].

In this study, we aimed to validate the clinical relevance of the 16-gene and 5-gene score methods in a Korean population. One of the key motivations of this study was that the genomic profiles of HCC patients were distinct between Western and Korean populations. For example, the *TP53* mutation rate is very high in Korean populations, but unlike African or Chinese populations, the *TP53* R249S mutation rate is almost zero and has no association with tumor recurrence and survival [5, 8, 15, 16]. In addition, genetic aberrations in *RB1* are associated with poor prognosis in Korean HCC patients, which has not been observed in Western populations [5, 8]. In this study, we generated the transcriptomic signatures using the 16-gene and 5-gene score methods, and analyzed them with the genomic profiles obtained by whole-exome sequencing and clinicopathological features of the HCC cases used.

## Methods

### Study design and clinical samples

Two hundred five Fresh frozen tissues of HCC patients at a surgically curable stage were used in this study. The surgically curable stage was defined by Milan criteria which clearly excludes extra hepatic metastasis and macrovascular invasion. Patients with either one of them cannot have surgical resection. The institutional review board of ASAN, South Korea, approved all of the samples, along with documented consent from all patients who participated in the study (2012–0389). The estimated tumor cellularity of each sample was more than 70%. Clinical features include tumor size, microvascular invasion, recurrence, the Edmondson-Steiner histological grade, the fibrosis stage of the non-neoplastic liver tissue, viral infection, tumor nodules (monofocal vs multifocal) and the serum alpha-fetoprotein levels (Table 1).

**Table 1:** Patients and tumor characteristics

Characteristics	Frequencies
Age at Surgery (Median, Range)	55 (26–80)
< 60 years	137 (66.83%)
> 60 years	68 (33.17%)
Etiology of the liver disease	
HBV^a^	148 (72.20%)
HCV^b^	20 (9.76%)
NBNC^c^	37 (18.05%)
Tumor size (median, range)	3.8 (1.2–16)
< 5 cm	140 (68.29%)
> 5 cm	68 (33.17%)
Microvascular invasion	
Yes	59 (28.78%)
No	146 (71.22%)
Serum AFP level (median, range)	42.7 (0.76–472,000)
< 20	91 (44.39%)
> 20	114 (55.61%)
Hepatic fibrosis stage	
1,2,3	109 (53.17%)
4	96 (46.83%)
Edmondson-Steiner grade	
1,2	137 (66.83%)
3,4	68 (33.17%)
Tumor nodules	
Single	193 (94.15%)
Multiple	12 (5.85%)

^a^HBV: hepatitis B virus.

^b^HCV: hepatitis C virus.

^c^NBNC: non-hepatitis B, non-hepatitis C

### Whole exome sequencing and copy number variation (CNV) analysis

As mentioned in our previous study, DNeasy Blood and Tissue kit (Qiagen) were used for DNA extraction from the tumor tissues. Exome sequencing was done using Illumina HiSeq 2000 platform [5]. The sequenced reads were aligned to the UCSC hg19 release of the human genome. Somatic mutations in *TP53*, *CTNNB1* and *RB1* were identified using MuTect [17]. CNV analysis was performed using the Affymetrix Cytoscan HD platform. CNV data were analysed with the Nexus Copy Number software (BioDiscovery, CA, ver. 6.1). Furthermore, LOH events were identified using PSCBS algorithm [18].

### G1-G6 classification and 5 gene score prediction

The detailed information about the development of G1-G6 and 5-gene score method was described in previous HCC studies [13, 14]. Briefly, the G1-G6 classification was done using robust unsupervised hierarchical clustering using 6712 probes of Affymetrix HG-U133A GeneChip™ [13]. The stability and reproducibility of the clusters were carefully evaluated. The mean reproducibility of all groups was more than 90%. On the basis of clusters, tumors were classified into G1-G6 subgroups. Furthermore, using quantitative RT-PCR data and applying 5 prediction algorithms (including SVM, PAM, kNN, DQDA, DLDA) on 103 genes associated with the prognosis and diagnosis of cancer, 16 genes were identified which properly classified HCC patients into G1-G6 subgroups. Similarly, univariate Cox model was generated for each of the 103 genes against prognosis and survival of HCC patients. 31 significant genes were further optimized to only 5 genes using multivariate COX model [14]. In this study, we evaluated the performance of G1-G6 and 5-gene scores in Korean population using high quality RNA data 205 of HCC patients.

### Statistical analysis

We performed several statistical analyses against these two molecular markers to establish their clinical and pathological relevance in a Korean population. IBM SPSS Version 20 was used for all statistical analyses. Fisher’s exact test (*P* < 0.05) was used to calculate any association between the genomic data and the G1-G6 and 5-gene score. Kaplan-Meier survival analysis was performed using DSF and RFS data. Previously, early recurrence was defined as recurrence before 24 months [14]. However, in our study recurrence before 12 months was considered as early recurrence. Univariate and multivariate Cox regression survival analyses were performed to validate the prognostic association between clinical data, genomic data and G1-G6 and 5-gene score.

## Results and discussion

Out of 231 cases of HCC used in previous study, we were able to extract high-quality RNA of 205 HCC cases [5]. We then found the association of G1-G6 subgroups and 5-gene score with mutations, CNVs and clinicopathological features (see Table 1 for clinical features).

### Validation of the G1-G6 classification in Korean population

**Fig. 1:**
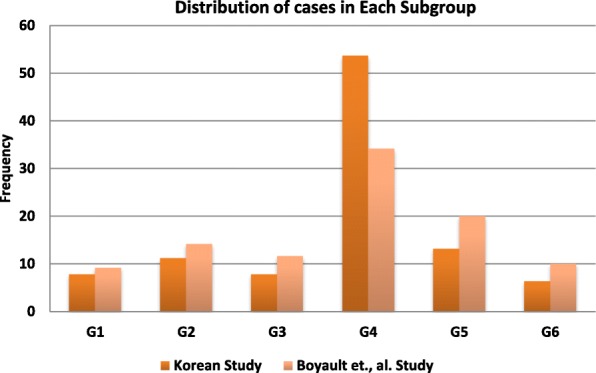
Distribution of G1-G6 subgroups in French and Korean studies

Out of the 205 cases analyzed, 16 (7.81%) fell into G1, 23 (11.22%) into G2, 16 (7.81%) into G3, 110 (53.65%) into G4, 27 (13.17%) into G5 and 13 (6.34%) into G6. The occurrence of the G1-G3 and G5-G6 subgroups in a previous French study were slightly higher compared to our dataset, except for the G4 subgroup, which accounted for the largest proportion in both studies (Fig. 1) [13, 14]. Of note, there is no significant difference between G1-G6 distribution in both cohorts (Mann–Whitney test, *P* = 0.297). In addition, the two cohorts were strongly correlated (Spearman Correlation = 0.89, *P* = 0.033).

### Molecular and clinicopathologic characteristics correlated with the G1-G6 classification

Then, we analyzed the G1-G6 subgroups with genetic aberrations and the clinical features of the 205 cases of HCC. G2 and G3 were significantly associated with *TP53* mutations (*P* < 0.005) and G5 and G6 with *CTNNB1* mutations (P < 0.005). Of note, almost all of the G6 subgroup cases (12/13) harbored somatic *CTNNB1* mutations. The associations between G2-G3 and *TP53* mutations and between G5-G6 and *CTNNB1* mutations in this study were consistent with the original observations made in the French study [13].

As for CNVs, G2 was significantly associated with 13q LOH (*P* < 0.05); G2-G3 with 17p LOH (*P* < 0.05); and G1-G3 with 4q (*P* < 0.001), 5q (*P* < 0.001) and 16p LOH (*P* < 0.001). All of these LOH events were associated with G1-G3, which are collectively recognized as HCC subgroups with chromosomal instability [13]. Again, the observations in this study were consistent with the original observations made in the French study [13].

As for clinicopathological features, G1-G3 groups were significantly associated with AFP > 100 IU/ml (*P* < 0.001), whereas, G4-G6 were significantly associated with AFP < 100 IU/ml (*P* < 0.001). G5 was significantly associated with tumor size (> 5 cm) (*P* < 0.05), which was not observed in the previous study. When we performed a survival analysis, the G1-G6 subgroups did not show any significant difference in either DSS or RFS (i.e., recurrence before 12 months), which was consistent with the French study [13].

### 5-gene score and its role in prognostication

**Fig. 2:**
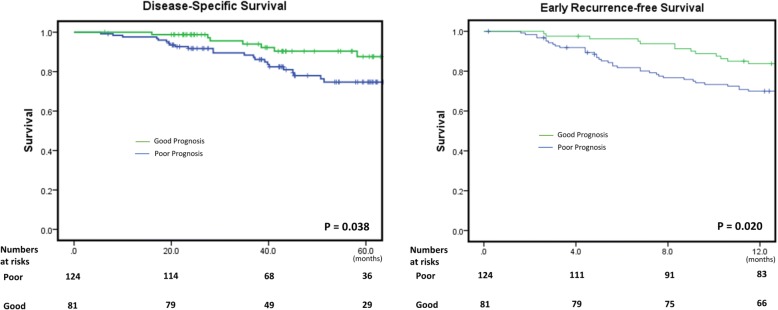
The good and poor prognosis groups, predicted by 5-gene score, show a significant difference in both DSS and early RFS

To validate the 5-gene score, we classified patients into a good prognosis group (81 cases, 40%) and a poor prognosis group (124 cases, 60%) according to the 5-gene score and evaluated the difference in DSS and RFS. As demonstrated in Fig. 2, the poor prognosis group showed a shorter median DSS (*P* < 0.05) and RFS (*P* < 0.05).

### Molecular and clinicopathologic features correlated with the 5-gene score

We found that the poor prognosis group, as predicted by the 5-gene score, was significantly associated with *TP53* mutations (*P* < 0.005). This association needs to be interpreted with caution. In our previous study, we reported that *TP53* mutations were not associated with poor survival in a Korean population; however, in other studies, *TP53* mutations, especially the R249S mutation resulting from aflatoxin B1 exposure, were associated with poor survival in HCC patients. In the 205 cases of HCC that we used in this study, no case harbored the *TP53 R249S* mutation. However, in the French study, 12 out 62 patients showed R249S mutations, all in migrants from Africa or Asia, but did not show any association with either survival or poor prognosis as predicted by the 5-gene score [14].

In addition, in our recent HCC study, we reported that *RB1* aberrations (Homozygous Deletions and Inactivating Mutations) were associated with the poor prognosis of HCC patients after resection. Consistent with previous observations, the poor prognosis predicted by the 5-gene score also showed association with *RB1* aberrations (i.e., 13 out of 18 (72%) cases fell into the poor prognosis group). In addition, the poor prognosis group was also significantly associated with a loss of heterozygosity (LOH) events at the 4q, 5q, 16p, 17p and 22q chromosomal arms, which was also consistent with the French study (*P* < 0.05) [13].

As for clinical features, the poor prognosis group was significantly associated with microvascular invasion (*P* < 0.005), tumor size (> 5 cm) (*P* < 0.05), and high AFP levels (> 20 ng/ml) *P* < 0.005). In addition, we found that the poor prognosis group was significantly associated with G1-G3 and the good prognosis group with G4-G6 (*P* < 0.005).

### Univariate and multivariate analysis for disease-specific survival (DSS)

With a median follow-up period of 53.3 months (range 5.5–88.9 months), 30 patients died due to HCC. The two- and five-year DSS rates were 94.5% and 79.9%, respectively. In the univariate analysis, DSS was significantly associated with the 5-gene score (hazard ratio (HR) 2.381, 95% confidence interval (CI) 1.021–5.56, *P* = 0.045), tumor nodules (HR 8.69, 95% CI 3.846–19.6653, *P* < 0.001), microvascular invasion (HR 2.496, 95% CI 1.218–5.118, *P* = 0.012), tumor size (HR 2.674, 95% CI 1.297–5.512, *P* = 0.008) and *RB1* aberrations (HR 3.133, 95% CI 1.344–7.303, *P* = 0.008) (Table 2). In the multivariable analysis, the 5-gene score was not validated as a significant prognostic factor; tumor nodules (HR 8.411, CI 3.263–21.681, *P* < 0.001) and tumor size (HR 2.534, CI 1.116–5.755, *P* = 0.026) were the significant prognostic factors for the survival of HCC patients after resection (Table 2).

**Table 2:** Cox regression analysis for DSS in 205 patients with resectable HCC

	Univariate analysis				Multivariable analysis			
			95.0% CI				95.0% CI	
	*P*-value	HR	Lower	Upper	*P*-value	HR	Lower	Upper
5-gene score (poor vs. good group)	0.045	2.381	1.021	5.551	0.278	1.368	0.518	3.614
Age (> = 60 vs. < 60)	0.867	1.033	0.706	1.511				
HBV	0.593	0.813	0.380	1.737				
HCV	0.076	1.548	0.956	2.507				
NBNC	0.547	0.906	0.658	1.249				
Tumor nodules	0.000	8.697	3.846	19.665	0.000	8.411	3.263	21.68
Cirrhosis (Yes vs. No)	0.581	1.225	0.597	2.515				
VI (Yes vs. No)	0.012	2.496	1.218	5.118	0.580	1.260	0.556	2.856
Tumor size (> = 5 cm vs < 5 cm)	0.008	2.674	1.297	5.512	0.026	2.534	1.116	5.755
Edmonson Grade	0.226	1.254	0.87	1.808				
AFP level (Normal (< 20) vs. elevated)	0.092	1.956	0.896	4.272				
RB1 mutation	0.008	3.133	1.344	7.303	0.527	1.368	0.518	3.614
TP53 mutation	0.629	0.819	0.364	1.841				
CTNNB1 mutation	0.212	0.542	0.207	1.418				

*VI Microvascular Invasion

### Univariate and multivariable analysis for early recurrence-free survival (RFS)

During follow-up, 49 out of 205 patients had disease recurrence within 12 months. In the univariate analysis, the early RFS was significantly associated with the 5-gene score (HR 2.087, 95% CI 1.106–3.936, *P* = 0.023), tumor nodules (HR 5.308, 95% CI 2.474–11.390, *P* < 0.001), HCV (HR 1.476, CI 1.010–2.156, *P* = 0.044), vascular invasion (HR 2.131, 95% CI 1.210–3.754, *P* = 0.009), AFP 20 ng/ml (HR 3.192, CI 1.631–6.248, *P* = 0.001), tumor size (HR 2.053, 95% CI 1.169–3.604, *P* = 0.012) and *RB1* aberrations (HR 2.62, 95% CI 1.302–5.228, *P* = 0.009) (Table 3). In the multivariate analysis, the 5-gene score was not validated as a significant prognostic factor; AFP20ng/ml (HR 2.918, CI 1.423–5.984, *P* = .003), tumor nodules (HR 6.818, CI 2.873–16.184, *P* < 0.001), HCV (HR 1.718, CI 1.159–2.546, *P* = .007) and tumor size (HR 1.909, CI 0.984–3.705, *P* = .056) were found as the significant prognostic factors for early RFS of HCC patients after resection (Table 3).

**Table 3:** Cox regression analysis for eary RFS in 205 patients with resectable HCC

	Univariate analysis				Multivariable analysis			
			95.0% CI				95.0% CI	
	*P*-value	HR	Lower	Upper	*P*-value	HR	Lower	Upper
5-gene score (poor vs. good group)	0.023	2.087	1.106	3.936	0.361	1.373	0.696	2.708
Age (> = 60 vs. < 60)	0.469	1.113	0.833	1.488				
HBV	0.814	1.079	0.572	2.035				
HCV	0.044	1.476	1.010	2.156				
NBNC	0.100	0.772	0.567	1.051				
Tumor Nodules	0.000	5.308	2.474	11.390	0.000	5.956	2.554	13.892
Cirrhosis (Yes vs. No)	0.223	1.418	0.809	2.485				
VI (Yes vs. No)	0.009	2.131	1.21	3.754	0.904	0.959	0.483	1.901
Tumor size (> = 5 cm vs < 5 cm)	0.012	2.053	1.169	3.604	0.058	1.885	0.978	3.634
Edmonson Grade	0.985	0.997	0.74	1.344				
AFP level (Normal (< 20) vs. elevated)	0.001	3.192	1.631	6.248	0.004	2.823	1.387	5.745
RB1 mutation	0.009	2.62	1.27	5.413	0.488	1.318	0.604	2.873
TP53 mutation	0.769	1.094	0.602	1.987				
CTNNB1 mutation	0.338	0.702	0.341	1.447				

*VI Microvascular Invasion

In this study, we aimed to validate the association of the G1-G6 signature and the prognostic value of the 5-gene score in Korean HCC patients. These two molecular signatures showed remarkable concordance between CNV and the mutation profiles of Korean HCC patients and the patients in French studies [13, 14], except for minor discrepancies. For example, G5 and G6 rates are lower in our cohort than in the original cohort, which seems to be related to the lower rate of *CTNNB1* mutation in our cohort. According to the 5-gene score, the poor prognosis group showed shorter disease-specific survival and early recurrence-free survival as well as a significant association with microvascular invasion, tumor size, high AFP levels, and *TP53* mutations.

However, the 5-gene score was not an independent prognostic factor for the survival of HCC patients. This may be due to the low event rate [only 30 patients out of 205 (14.6%) died during follow-up, in contrast to French data, in which 106/314 (33.8%) died during follow-up], which may have resulted in different multivariable outcomes.

## Conclusions

Thus, our analysis suggests that G1-G6 and 5-gene signatures are in concordance between genetic profiles of Korean HCC patients and patients in original French studies. Therefore, in the future, by combining all of these cohorts, we may be able to assertively establish the clinical and pathological relevance of the 5-gene score and develop therapeutic strategies for HCC patients worldwide.

## Abbreviations

CNV: Copy Number Variation
DSS: Disease-Specific Survival
HCC: Hepatocellular Carcinoma
LOH: Loss Of Heterozygosity
RFS: Recurrence-Free Survival
